# Effect of Washed Microbiota Transplantation on Patients With Dyslipidemia in South China

**DOI:** 10.3389/fendo.2022.827107

**Published:** 2022-04-22

**Authors:** Fenfen Liang, Xinjian Lu, Zhiliang Deng, Hao-Jie Zhong, Wei Zhang, Qing Li, Hong-Hao Zhou, Yu-Ligh Liou, Xing-Xiang He

**Affiliations:** ^1^ Department of Gastroenterology, The First Affiliated Hospital of Guangdong Pharmaceutical University, Guangzhou, China; ^2^ Department of Gastroenterology, Research Center for Engineering Techniques of Microbiota-Targeted Therapies of Guangdong Province, Guangzhou, China; ^3^ Department of Clinical Pharmacology, Xiangya Hospital, Central South University, Changsha, China; ^4^ Clinical Precision Medicine Research Center, The First Affiliated Hospital of Guangdong Pharmaceutical University, Guangzhou, China; ^5^ Xiangya Medical Laboratory, Central South University, Changsha, China

**Keywords:** fecal microbiota transplantation, washed microbiota transplantation (WMT), hyperlipidemia, hypolipidemia, blood lipid

## Abstract

**Background and Aims:**

Although the manual crude fecal microbiota transplantation (FMT) reduces blood lipids in animal models of hyperlipidemia, its clinical effect on blood lipid metabolism in patients with hyperlipidemia and hypolipidemia remains unclear, especially in the Chinese population. It was reported that washed microbiota transplantation (WMT) was safer, more precise, and more quality-controllable than the crude FMT by manual. This study aimed to investigate the feasibility and effectiveness of WMT on lipid metabolism in the Chinese population.

**Methods:**

Clinical data of patients with various indications who received WMT for 1–3 treatment procedures were collected. Changes in blood lipids before and after WMT, namely, total cholesterol (TC), triglyceride (TG), high-density lipoprotein cholesterol (HDL-C), low-density lipoprotein cholesterol (LDL-C), homeostasis model assessment of insulin resistance (HOMA-IR), liver fat attenuation, and liver stiffness measurement, were compared.

**Results:**

A total of 177 patients (40 cases of hyperlipidemia, 87 cases with normal blood lipids, and 50 cases of hypolipidemia) were enrolled in the First Affiliated Hospital of Guangdong Pharmaceutical University. WMT has a significant therapeutic effect in reducing blood lipid levels (TC and TG) in the short- and medium term in patients with hyperlipidemia (p <0.05). Hyper blood lipid decreased to normal in the short-term (35.14%; p <0.001), and LDL-C changed to normal in the medium term (33.33%; p = 0.013). In the hypolipidemia group, 36.36% and 47.06% changed to normal in the short-term (p = 0.006) and medium term (p = 0.005) of therapeutic effects based on blood lipid levels. In the normal blood lipid group and the low-risk group of atherosclerotic cardiovascular disease (ASCVD), the change was not statistically significant, indicating that WMT does not increase the risk of blood lipid and ASCVD in the long-term.

**Conclusions:**

WMT treatment changes blood lipids in patients with hyperlipidemia and hypolipidemia without serious adverse events, with no risk for increasing blood lipids and ASCVD in the long-term. There were significant decreased TC, TG, and LDL-C levels in the medium term of WMT treatment for hyperlipidemia. Therefore, the regulation of gut microbiota by WMT may indicate a new clinical method for the treatment of dyslipidemia.

## Introduction

Cardiometabolic syndrome (CS), also known as metabolic syndrome (MS), is caused by a series of metabolic diseases related to pathogenesis, namely, hyperlipidemia, hypertension, diabetes, and obesity. The presence of the combined effects of these metabolic diseases can significantly increase the risk of developing cardiovascular disease. We are entering an era of chronic metabolic and cardiovascular multi-comorbidities. It is estimated that 22% of the adult population in the US suffers from CS, and its prevalence is on the rise (1). Previous studies have proven that hyperlipidemia is a severe risk factor for coronary artery disease (CAD), which accounts for approximately 30% of deaths worldwide (2). The treatment strategy for hyperlipidemia is mainly lifestyle intervention and drug therapy. However, pharmacologic therapies are associated with significant side effects with long-term use (3).

In China, atherosclerotic cardiovascular disease (ASCVD) has become the first cause of death, and dyslipidemia is the most important pathogenic risk factor in the occurrence and development of ASCVD (4). According to the data of 29,678 Chinese adults aged ≥35 years from 2012 to 2015 (5), the prevalence of dyslipidemia was 34.7% higher in men than in women (40.0% vs. 29.3%; p <0.001), and there was no significant difference between urban and rural residents (35.7% vs. 34.1%). In recent years, the mortality rate of CAD in China has been increasing, and the first reason is the increase in cholesterol (77%). Hyperlipidemia has become an important public health problem. In addition, high-density lipoprotein cholesterol (HDL-C) hypolipidemia is associated with many kinds of CS, and has displayed an increasing prevalence in China (6). Therefore, it is of great significance to comprehensively analyze the related factors of hyperlipidemia and find a treatment method with less side effects.

Gut microbiota disorder is a risk factor for CS (1, 7). Fecal microbiota transplantation (FMT) is a new therapeutic method, which uses “healthy” microbial configuration to replace the indigenous microbiome of patients. To date, FMT has been used in the clinical application of recurrent *Clostridium difficile* disease (CDI) infection and has been studied in other microbiome-associated diseases, such as ulcerative colitis (UC), inflammatory bowel disease (IBD), diabetes, and cardiac metabolic syndrome (8). Whether FMT can improve cardiac metabolic syndrome is a subject to be discussed in clinical medicine.

Given the safety, quality-control, and effective profile of washed microbiota transplantation (WMT) on microbiota disorder disease, we attempted to investigate the patients receiving WMT to observe whether WMT can improve human metabolism and blood lipid profiles (9, 10). We hypothesized that WMT could safely and continuously affect patients with various indications and change dyslipidemia without side effects. Therefore, we conducted a retrospective trial to collect the medical data of WMT-treated patients with dyslipidemia (hyperlipidemia and hypolipidemia).

## Materials and Methods

### Patients

This study was conducted and approved by the Ethics Committee (no. 2017-98), in accordance with the Declaration of Helsinki at the First Affiliated Hospital of Guangdong Pharmaceutical University, Guangzhou, China. Written informed consent from all patients was obtained and reviewed. Consecutive patients who underwent WMT (9) for various indications with 1–3 courses at our hospital from January 1, 2017 to June 31, 2020 were enrolled in the study. The inclusion criteria were as follows: patients who were >18 years old, those who provided informed consent, and those who received WMT. The exclusion criteria were as follows: patients who were pregnant, those who had antibiotics and probiotics 3 months before and during the study, and those who had a change in their antihyperlipidemia medication regimen after WMT. The minimum sample size was 12 allogeneic patients based on the individual patient data from Vrieze et al. (11) and the calculation method from Craven et al. (12).

### WMT Procedure

The procedure of WMT was consistent with the Nanjing Consensus on Methodology of Washed Microbiota Transplantation (10). All healthy stool donors, aged between 18 and 25 years old, strictly underwent interviews, psychological, and physical examinations, biochemical testing, and infectious disease screening. Briefly, the microbiota for WMT was extracted from donated feces followed by centrifugation and suspension for three times using the intelligent microbial separation system (GenFMTer; FMT Medical, Nanjing, China), and five stages of filtration according to the recommendations of the manufacturer. Thereafter, a fecal suspension is injected into the body of the patient through a nasojejunal tube (upper digestive tract) or an endoscopic intestinal tube (lower digestive tract) once a day for 3 days of one WMT course (13). The standard treatment timelines of the three WMT courses in our center were 0, 1, and 2 months respectively, and the end-point return visit time was 4 months. All patients underwent at least one WMT procedure and completed follow-up.

### Data Collection

Data were collected from the medical records of patients at prior treatment (baseline), before the second procedure (short-term effect), before the third procedure (medium term effect) and the clinical visit at the 4 months (long-term effect). Data included were age, sex, body mass index (BMI), smoking status, blood pressure (at admission), diseases or indications for WMT, and laboratory test results, namely, serum lipids (total cholesterol [TC], triglyceride [TG], high-density lipoprotein [HDL-C], and low-density lipoprotein [LDL-C]), fasting glucose (FG) and fasting insulin (FI), liver fat attenuation, and liver stiffness measurement (LSM). Homeostasis model assessment of insulin resistance (HOMA-IR) value was calculated as previously described (14). Hyperlipidemia was diagnosed based on the guideline for dyslipidemias (4, 15). All eligible patients are divided into three groups: the hyperlipidemia (TC ≥6.2 mmol/L or TG ≥2.3 mmol/L or LDL-C ≥4.1 mmol/L), hypolipidemia (TC <3.6mmol/L or TG <0.33 mmol/L or LDL-C <2.07 mmol/L or HDL <0.91) by hospital definition, and the normal groups according to their baseline, blood lipid status, and risk stratification of ASCVD. The diagnostic criterion of abnormal risk factors for HOMA-IR is ≥2.5 (16). The diagnostic threshold of fat attenuation is mild (≥240 db/m), moderate (≥266 db/m), and severe (≥295 db/m). LSM diagnostic threshold is >14.1 kPa (17). Diabetes (≥7.0), impaired glucose tolerance (<7.0), impaired fasting blood glucose (6.1–7.0), and normal (4.4–7.0) are defined by fasting blood glucose level (mmol/L) (18). Obesity (≥28.0), overweight (24.0–28.0), normal weight (18.5–24.0), and underweight (<18.5) are classified according to the definition of BMI (kg/m^2^) (19).

All patients were divided into three groups according to the above definition. After all patients received 1–3 times of WMT treatment procedure and completed follow-up, the results of blood lipid changes were statistically analyzed and evaluated.

### Statistical Analysis

Statistical analyses were performed using SPSS 22.0 (IBM Corp., Armonk, NY, USA). Results were expressed as frequencies and percentages for categorical variables, means and standard deviations for normally distributed continuous variables, and medians and interquartile ranges for non-normally distributed continuous variables. Categorical variables were analyzed using the Chi-square or Fisher exact test. For comparisons of continuous variables between two independent groups, either unpaired Student’s t-test (for normally distributed variables) or Mann–Whitney U test (for non-normally distributed variables) was applied. While comparing paired data, paired Student’s t-test (for normally distributed variables) or Mann–Whitney U test (for non-normally distributed variables) was used. A two-tailed P-value <0.05 was considered statistically significant.

## Results

### Clinical Characteristics of Patients Who Underwent WMT

**Figure 1:**
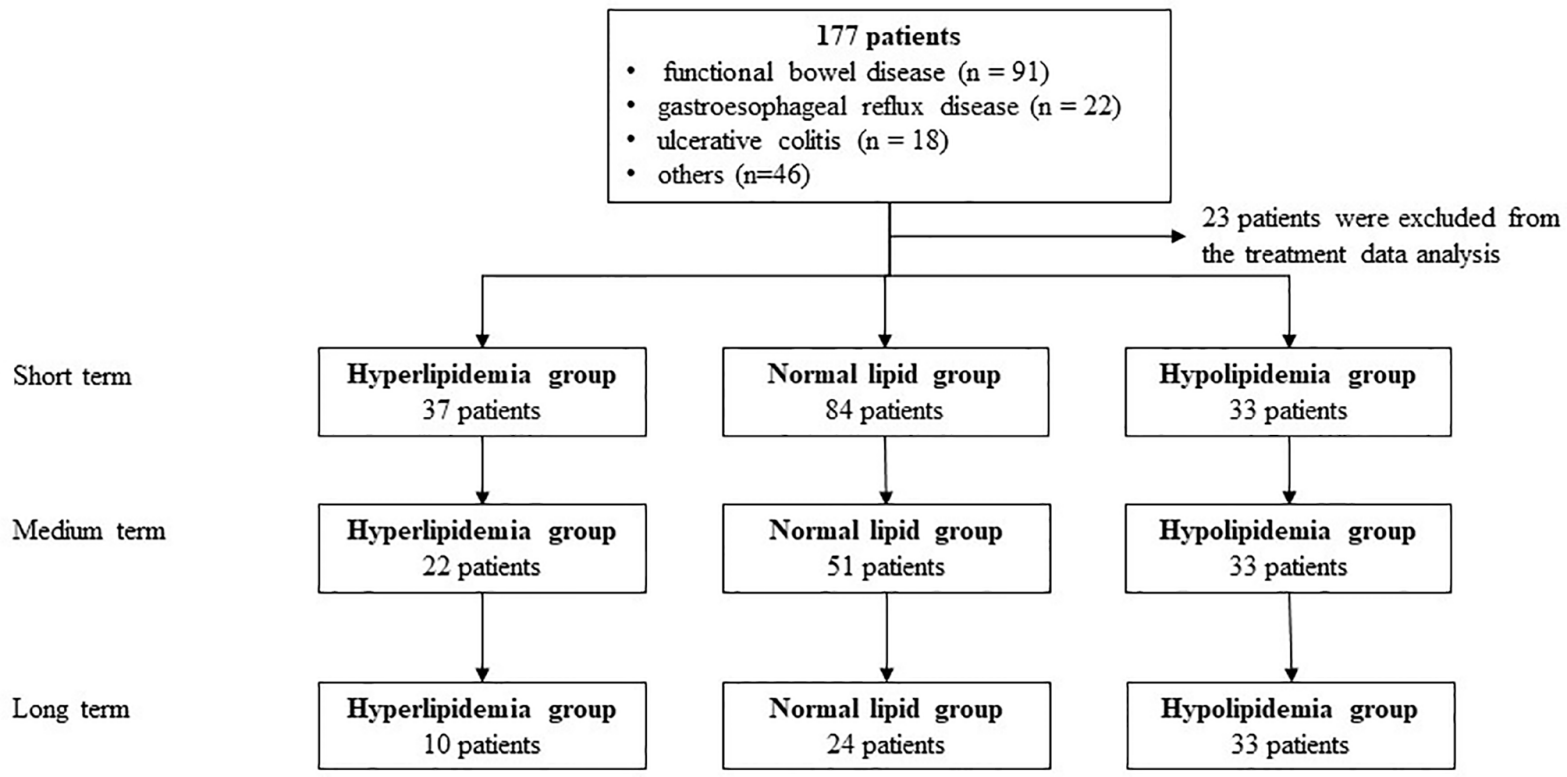
The Flow Chart of the Study.

A total of 177 patients (40 cases of hyperlipidemia, 50 cases of hyperlipidemia, and 87 cases with normal lipid) were treated with WMT, of which 52.0% were male, with an average age of 52.77 ± 15.79 years (**Figure 1**). **Table 1** shows the main indications for WMT, and the top three diseases were functional bowel disease (n = 91; 51.4%), followed by gastroesophageal reflux disease (n = 22; 12.4%) and ulcerative colitis (n = 18; 10.2%). The interval time of 3 treatment procedures were 37 days (33–47) for short-term, 41days (34–63) for medium term, and 96 days (77–119) for long-term. Blood lipid characteristics of patients are illustrated in **Table 2**. The average BMI of the hyperlipidemia group was classified as overweight, while the other two groups were normal. The average TG of the hyperlipidemia group was 2.66 (1.37–3.24) mmol/L higher, but TC and LDL-C were lower than the definition described in *Materials and Methods*. The average TC and LDL-C of the hypolipidemia group were 3.59 (3.34–3.83) mmol/L and 1.97 ± 0.65 mmol/L, which was lower than the definition of hypolipidemia. The average TC, TG, LDL of the normal group was 4.64 (4.29–5.15), 0.97 (0.72–1.19), and 2.93 ± 0.528 mmol/L. The average of HOMA-IR of the hyperlipidemia group is 2.71 (1.51–3.76), which was higher than the diagnostic threshold, and the others were in the normal range. The average of liver fat attenuation of hyperlipidemia and hypolipidemia groups were mild (251.00 [242.00–279.25]) and moderate (282.00 [259.50–287.75] db/m). There are no serious adverse events (AEs), and only a few of AEs include abdominal pain, abdominal distention, diarrhea, and dizziness.

**Table 1:** Main diagnoses of patients receiving washed microbiota transplantation.

Diagnoses	Number (n)	Percentage (%)
Functional bowel disorders	91	51.4%
Gastroesophageal reflux disease	22	12.4%
Ulcerative colitis	18	10.2%
Nonalcoholic fatty liver disease	17	9.6%
Hyperuricemia	8	4.5%
HBV-related liver cirrhosis	8	4.5%
Radioactive enteritis	2	1.1%
Hepatic encephalopathy.	2	1.1%
Intestinal tuberculosis	1	0.6%
Epilepsy	1	0.6%
Colon carcinoma	1	0.6%
Crohn's disease	1	0.6%
Senile tremor	1	0.6%
Parkinson's disease	1	0.6%
Bipolar disorder	1	0.6%
Gastric malignant tumor	1	0.6%
Autoimmune hepatitis	1	0.6%
Total	177	

HBV, Hepatitis B virus.

**Table 2:** Demographics and clinical characteristics of patients at baseline.

	Hyperlipidemia group (n = 40)	Normal lipid group (n = 87)	Hypolipidemia group (n = 34)
Age (years)	53.20 ± 12.66	53.44 ± 16.36	52.76 ± 17.47
Male n (%)	18 (45)	37 (42.5)	28 (82.4)
BMI	25.07 (21.82–28.34)	22.32 (19.53–25.07)	21.62 (19.19–23.38)
	(n = 37)	(n = 87)	(n = 34)
FG (mmol/L)	4.69 (4.34–5.52)	4.58 (4.18–5.03)	4.56 (4.04–5.00)
	(n = 37)	(n = 80)	(n = 32)
FI (mmol/L)	9.79 (6.78–16.56)	7.81 (5.15–11.63)	6.93 (4.99–9.47)
	(n = 28)	(n = 65)	(n = 22)
TC (mmol/L)	5.91 (5.26–6.36)	4.64 (4.29–5.15)	3.59 (3.34–3.83)
TG (mmol/L)	2.66 (1.37–3.24)	0.97 (0.72–1.19)	0.92 (0.57–1.17)
LDL-C(mmol/L)	3.36 ± 1.13	2.93 ± 0.53	1.97 ± 0.65
HDL-C(mmol/L)	1.22 (1.03–1.39)	1.14 (1.37–1.60)	1.11(0.88–1.41)
HOMA-IR	2.71 (1.51–3.76)	1.62 (1.08–12.59)	1.57(1.01–2.53)
	(n = 27)	(n = 60)	(n=22)
liver fat attenuation	251.00 (242.00–279.25)	237.00 (215.00–298.50)	282.00(259.50–287.75)
	(n = 20)	(n = 22)	(n=6)
liver stiffness measurement	6.55 (5.28–8.50)	7.15 (5.25–8.65)	8.40(6.63–9.63)
	(n = 20)	(n = 22)	(n=6)

Data presented as mean ± standard deviation, median (interquartile range), or n (%).

BMI, body mass index; FG, fasting glucose; FI, fasting insulin; TC, total cholesterol; TG, triglyceride; HDL-C, high-density lipoprotein cholesterol; LDL-C, low-density lipoprotein cholesterol; HOMA–IR, homeostasis model assessment of insulin resistance.

### Effect of WMT on Lipid Profiles

**Figure 2:**
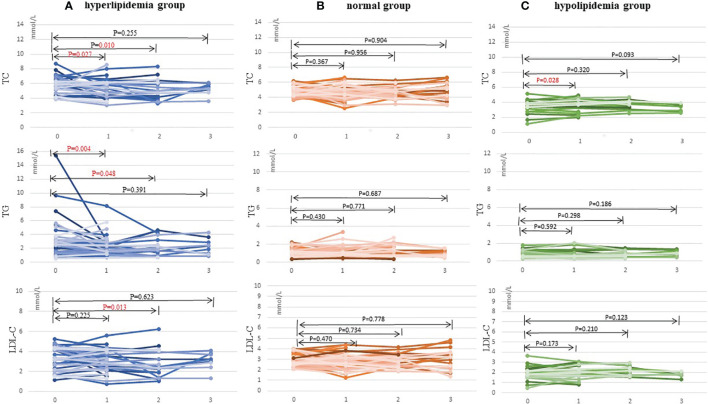
Changes in TC, TG, and LDL-C levels after 1–3 times of washing microbial transplantation. **(A)** Changes in TC, TG, and LDL-C levels in the hyperlipidemia group; **(B)** Changes in TC, TG, and LDL-C levels in the normal group; and **(C)** Changes in TC, TG, and LDL-C levels in the hypolipidemia group. TC, total cholesterol; TG, triglyceride; LDL-C, low-density lipoprotein cholesterol.

**Table 3** and **Figure 2** show the effect of WMT on hyperlipidemic, normal, and hypolipidemic lipid profiles. In the short-term, WMT showed a significantly lower effect on TC (from 5.81 ± 1.13 to 5.26 ± 1.26 mmol/L) and TG (from 2.71 [1.45–3.46] to 1.76 [1.26–2.78] mmol/L) in the hyperlipidemia group (p <0.05), and a significantly higher effect on TC (from 3.58 [3.21–3.82] to 3.74 [3.32–4.44]) in the hypolipidemia group (p <0.05). In the medium term, WMT also showed a significantly lower effect on TC (p = 0.01), TG (p = 0.048), and LDL-C (p = 0.013) in the hyperlipidemia group (p <0.05). In the hyperlipidemia group, WMT explained the short-term blood lipid reduction effect on TC and TG, and mid-term blood lipid reduction effect on TC, TG, and LDL-C. In hyperlipidemia group, TC, TG, and LDL-C except HDL-C (p = 0.024) had no significant change in the long-term effect of WMT. Since the number of long-term monitoring is only 9, it cannot be confirmed whether WMT treatment has long-term effects in the study. In the hypolipidemia group, the WMT treatment caused an increase in TC, LDL-C, and HDL-C levels from short to long-term, although all blood lipids results were not found significant in the long-term. WMT does not affect the changes in blood lipids of the normal group from short to long-term. There was no significant change in BMI before and after treatment in the three groups.

**Table 3:** The lipid profiles of short-, medium-, and long-term washed microbiota transplantation treatment procedures in the study.

Items	Baseline	Short-term	p-Value	Baseline	Medium-term	p-Value	Baseline	Long-term	p-Value
**Hyperlipidemia group**									
BMI	25.01 ± 3.52	24.50 ± 3.68	0.070	24.68 ± 3.51	23.95 ± 3.72	0.233	25.31±4.48	25.31 ± 4.98	0.999
	(n = 37)	(n = 37)		(n = 22)	(n = 22)		(n=10)	(n = 10)	
TC	5.81 ± 1.13	5.26 ± 1.26	0.027	6.03 ± 1.10	5.09 ± 1.37	0.010	5.56±0.82	5.27 ± 0.78	0.255
	(n = 37)	(n = 37)		(n = 21)	(n = 21)		(n=9)	(n = 9)	
TG	2.71 (1.45–3.46)	1.76 (1.26–2.78)	0.004	2.6 (1.49–3.13)	1.85 (1.12–2.82)	0.048	2.52 ±0.73	2.30 ± 1.1	0.391
	(n = 37)	(n = 37)		(n = 21)	(n = 21)		(n=9)	(n = 9)	
LDL-C	3.32 (2.24–4.25)	3.04 (2.26–3.92)	0.225	3.48 ± 1.10	2.89 ± 1.33	0.013	3.17±0.95	3.07 ± 0.84	0.623
	(n = 37)	(n = 37)		(n = 21)	(n = 21)		(n=9)	(n = 9)	
HDL-C	1.17 ± 0.29	1.23 ± 0.31	0.161	1.22 (1.11–1.40)	1.15 (1.03–1.43)	0.985	1.24±0.16	1.15 ± 0.18	0.024
	(n = 37)	(n = 37)		(n = 21)	(n = 21)		(n=9)	(n = 9)	
**Normal group**									
BMI	22.40 (19.53–25.10)	22.22 (19.63–25.38)	0.737	22.48 (19.56–25.71)	22.06 (19.40–25.65)	0.447	22.25 (18.81–26.67)	22.91 (19.75–25.42)	0.648
	(n = 84)	(n = 84)		(n = 51)	(n = 51)		(n=24)	(n = 24)	
TC	4.62 (4.28–5.11)	4.65 (4.23–5.09)	0.367	4.44 (4.26–5.00)	4.57 (4.03–4.91)	0.956	4.87±0.68	4.86 ± 1.10	0.904
	(n = 58)	(n = 58)		(n = 37)	(n = 37)		(n=19)	(n = 19)	
TG	1.00 (0.78–1.21)	0.99 (0.73–1.25)	0.430	0.97 (0.78–1.16)	0.89 (0.73–1.33)	0.771	0.94 (0.76–1.16)	0.91 (0.69–1.16)	0.687
	(n = 58)	(n = 58)		(n = 37)	(n = 37)		(n=19)	(n = 19)	
LDL-C	2.70 (2.52–3.13)	2.79 (2.24–3.15)	0.470	2.69 (2.49–3.10)	2.67(2.35–3.05)	0.734	3.14±0.56	3.08 ± 0.96	0.778
	(n = 58)	(n = 58)		(n = 37)	(n = 37)		(n=19)	(n = 19)	
HDL-C	1.32(1.13–1.61)	1.29 1.11–1.63)	0.151	1.30 (1.13–1.59)	1.27 (1.11–1.53)	0.142	1.36±0.32	1.33 ± 0.32	1.000
	(n = 58)	(n = 58)		(n = 37)	(n = 37)		(n=19)	(n = 19)	
**Hypolipidemia group**									
BMI	21.65 ± 3.73	21.48 ± 3.51	0.559	21.22 ± 3.93	21.48 ± 3.05	0.434	20.34±3.63	20.95 ± 0.99	0.249
	(n = 33)	(n = 33)		(n = 22)	(n = 22)		(n=11)	(n = 11)	
TC	3.58 (3.21–3.82)	3.74 (3.32–4.44)	0.028	3.63 (3.36–3.88)	3.73 (3.28–4.12)	0.320	3.30 (2.78–3.61)	3.52 (2.79–3.87)	0.093
	(n = 22)	(n = 22)		(n = 17)	(n = 17)		(n=8)	(n = 8)	
TG	0.84 (0.50–1.14)	0.69 (0.55–1.19)	0.592	0.90 ± 0.40	0.83 ± 0.36	0.298	0.80±0.40	0.94 ± 0.37	0.186
	(n = 22)	(n = 22)		(n = 17)	(n = 17)		(n=8)	(n = 8)	
LDL-C	1.91 ± 0.68	2.06 ± 0.67	0.173	1.82 (1.79–2.10)	2.07 (1.65–2.49)	0.210	1.79(1.53–1.90)	1.84 (1.73–2.10)	0.123
	(n = 22)	(n = 22)		(n = 17)	(n = 17)		(n=8)	(n = 8)	
HDL-C	1.15 ± 0.37	1.21 ± 0.35	0.054	1.20 ± 0.44	1.24 ± 0.33	0.602	1.05±0.35	1.20 ± 0.48	0.779
	(n = 22)	(n = 22)		(n = 17)	(n = 17)		(n=8)	(n = 8)	

presented as mean ± standard deviation, median (interquartile range), or n (%).

BMI, body mass index; TC, total cholesterol; TG, triglyceride; HDL-C, high-density lipoprotein cholesterol; LDL-C, low-density lipoprotein cholesterol.

### Effect of WMT on HOMA-IR, Liver Fat Attenuation, and LSM

Observation of WMT treatment effects from short-, medium- and long-term changes on FG, FI, HOMA-IR, liver fat attenuation, and LSM are shown in **Table 4**. After WMT treatment, abnormal HOMA-IR did not improve, and other indexes did not change significantly in the three groups. Only liver fat attenuation decreased significantly by WMT treatment in the long-term (p = 0.043).

**Table 4:** The glucose metabolism and liver profiles of short-, medium-, and long-term washed microbiota transplantation treatment in the study.

Items	Baseline	Short-term	p-Value	Baseline	Medium-term	p-Value	Baseline	Long-term	p Value
**Hyperlipidemia group**									
FG	4.66 (4.34–5.84	4.80 (4.34–6.05)	0.412	4.61 (4.24–5.24)	5.04 (4.25–6.04)	0.117	5.28 (4.61–8.28)	4.95 (4.85–1023)	0.672
	(n = 34)	(n = 34)		(n = 17)	(n = 17)		(n=7)	(n=7)	
FI	11.76 (6.78–16.60)	12.45 (8.02–18.29)	0.247	12.91 (7.44–17.34)	15.21 (13.47–22.72)	0.074	16.57 (7.81–20.92)	14.63 (9.84–23.08)	0.686
	(n = 20)	(n = 20)		(n = 10)	(n = 10)		(n=5)	(n=5)	
HOMA-IR	2.71 (1.51–3.76)	2.29 (1.61–4.15)	0.212	3.20 ± 1.44	5.31 ± 3.77	0.059	3.90±1.70	4.28±2.12	0.500
	(n = 19)	(n = 19)		(n = 10)	(n = 10)		(n=5)	(n=5)	
Liver fat attenuation	255.00 (242.00–281.00)	241.00 (226.00–282.00)	0.532	249.00 (235.50–284.50)	240.00 (226.50–287.0)	0.761	275.75±28.93	284.50±36.79	0.743
	(n = 15)	(n = 15)		(n = 9)	(n = 9)		(n=4)	(n=4)	
Liver stiffness measurement	6.80 (5.80–8.80)	6.60 (5.20–7.50)	0.256	5.80 (3.80–7.90)	6.30 (5.80–7.90)	0.192	5.40 (3.75–7.43)	6.20 (5.98–7.55)	0.465
	(n = 15)	(n = 15)		(n = 9)	(n = 9)		(n=4)	(n=4)	
**Normal group**									
FG	4.59 (4.18–5.07)	4.49 (4.24–4.87)	0.204	4.52 (4.068–5.05)	4.41 (3.91–4.90)	0.129	4.37 (3.99–5.17)	4.39 (4.04–5.11)	0.387
	(n = 69)	(n = 69)		(n = 42)	(n = 42)		(n=19)	(n=19)	
FI	8.41 (5.16–12.49)	8.23 (5.79–13.68)	0.596	8.56 (4.29–14.54)	9.04 (5.68–12.43)	0.673	9.71 (6.52–16.06)	7.81 (6.14–15.94)	0.347
	(n = 39)	(n = 39)		(n = 22)	(n = 22)		(n=12)	(n=12)	
HOMA-IR	1.65 (1.08–2.87)	1.96 (1.10–2.85)	0.530	1.70 (0.81–3.99)	1.98 (1.10–2.84)	0.809	1.85 (1.23–4.30)	1.49 (1.25–2.71)	0.114
	(n = 36)	(n = 36)		(n = 19)	(n = 19)		(n=10)	(n=10)	
Liver fat attenuation	260.39 ± 64.48	255.30 ± 57.77	0.372	281.23 ± 68.12	262.23 ± 50.48	0.100	240.40±28.90	229.60±38.53	0.043
	(n = 18)	(n = 18)		(n = 13)	(n = 13)		(n=5)	(n=5)	
Liver stiffness measurement	7.2 (5.63–8.65)	7.00 (5.90–10.43)	0.248	8.42 ± 5.03	8.25 ± 4.28	0.916	8.60(5.55–12.95)	8.10(6.50–13.20)	0.893
	(n = 18)	(n = 18)		(n = 13)	(n = 13)		(n=5)	(n=5)	
**Hypolipidemia group**									
FG	4.52 (4.00–4.96)	4.39 (3.86–5.25)	0.552	4.51 (3.97–5.20)	4.49 (4.11–5.30)	0.845	4.43(3.84–4.77)	4.51(3.73–5.08)	0.515
	(n = 29)	(n = 29)		(n = 18)	(n = 18)		(n=9)	(n=9)	
FI	6.77 (3.20–10.15)	8.26 (4.86–11.11)	0.372	7.71 (6.53–11.06)	6.07 (4.58–18.06)	0.889	9.43±6.75	8.48±2.17	0.809
	(n = 18)	(n = 18)		(n=8)	(n=8)		(n=4)	(n=4)	
HOMA-IR	1.57 (0.85–2.69)	1.73 (1.07–4.39)	0.122	2.42 (1.53–3.10)	1.65 (0.87–6.20)	0.674	1.90±1.37	1.97±1.49	0.918
	(n = 18)	(n = 18)		(n=8)	(n = 8)		(n=4)	(n=4)	
Liver fat attenuation	281.20 ± 11.14	263.60 ± 31.90	0.290	283.00	261.00	–	249.00	266.00	–
	(n = 5)	(n = 5)		(n=1)	(n = 1)		(n=1)	(n=1)	
Liver stiffness measurement	8.40 ± 2.02	11.32 ± 6.45	0.236	6.10	6.20	–	8.00	6.10	–
	(n = 5)	(n = 5)		(n=1)	(n = 1)		(n=1)	(n=1)	

Data presented as mean ± standard deviation, median (interquartile range), or n (%).

FG, fasting glucose; FI, fasting insulin; HOMA–IR, homeostasis model assessment of insulin resistance.

### Evaluation of WMT Clinical Therapeutic Effect on Lipid Levels

All enrolled patients are divided into hyperlipidemia, normal lipid, and hypolipidemia groups based on the based line of blood lipid. Patients were regrouped according to the changes of blood lipid levels after 1–3 WMT treatment procedures (**Table 5**). The three groups showed significant changes in blood lipid levels in short and medium terms. In the hyperlipidemia group, 35.14% recovered to normal in the short-term, and 33.33% recovered to normal in the medium term (p <0.005), and 40.00% in the long-term (p = 0.094). In the hypolipidemia group, 36.36% recovered to normal in the short-term, 47.06% recovered to normal in the medium term (p <0.005), and 12.5% in the long-term (p = 0.302). Our data also demonstrated that WMT could significantly alter the higher and lower lipid levels of patients to normal in the short and medium term of treatments; however, the efficacy of WMT remained to be explored. Patients with normal lipid levels changed to abnormal in the short and medium terms (25.86 and 20.00%, respectively; p <0.05) and in the long-term (27.78%; p = 0.054). Our data showed that WMT might have changed the blood lipid levels after one procedure treatment, while the lipid levels became steady in the medium and long-term treatments.

**Table 5:** Short-, medium-, and long-term therapeutic effects on blood lipid levels and risk stratification of atherosclerotic cardiovascular disease risk stratification.

Data periods	n	Therapeutic effect base on blood lipid levels				n	Therapeutic effect base on ASCVD risk stratification					
		Unchanged group (n)	Changed group (n)	X^2^	p-Value		Unchanged group (n)	Risk descended (n)		X^2^		p-Value
	**Hyperlipidemia group**					**High-risk group**						
Short-term	37	24	13	15.770	<0.001	10	6	4		2.813		0.094
Medium-term	21	14	7	6.171	0.013	4	2	2		0.667		0.414
Long-term	10	6	4	2.813	0.094	2	1	1		1.726		1.789
	**Hypolipidemia group**					**Medium-risk group**						
Short-term	22	14	8	7.486	0.006	15	10	5		3.840		0.050
Medium-term	17	9	8	8.010	0.005	9	4	5		4.431		0.035
Long-term	8	7	1	1.067	0.302	4	3	1		1.143		0.285
	**Normal group**					**Low-risk group**						
Short-term	58	43	15	17.228	<0.001	74	71	3		1.334		0.248
Medium-term	37	31	6	4.534	0.033	49	47	2		0.510		0.475
Long-term	18	13	5	3.716	0.054	21	21	4		2.446		0.118

The definition of unchanged and changed of hyperlipidemia group were still hyperlipidemia and changed to normal.

The definition of unchanged and changed of hypolipidemia group were still hypolipidemia and changed to normal.

The definition of unchanged and changed of normal group were still normal and changed to abnormal, including hyperlipidemia and hypolipidemia.

The definition of unchanged of high-, medium-, and low-risk groups were still risk, medium-, and low-risk after WMT procedures, respectively.

### Evaluation of the Therapeutic Effect of WMT on the Risk for ASCVD

Patients were divided into extremely-high-risk, high-risk, medium-risk, and low-risk groups according to the ASCVD risk stratification. Patients were regrouped into the no risk changed group and the risk-ascended group after WMT treatment procedures (**Table 5**). Patients with an acute coronary syndrome, stable coronary heart disease, postoperative revascularization, ischemic cardiomyopathy, ischemic stroke, transient ischemic attack, and peripheral atherosclerosis were included in the extremely high-risk group, and this group of patients will not be reassigned after WMT treatment.

No significant WMT therapeutic effect was found in short, medium, and long terms in the high-risk group. In the medium-risk group, 33.33% in the short-term and 55.56% in the medium term were regrouped to the low-risk group (p <0.05). In the low-risk group of ASCVD, the change was not statistically significant, indicating that WMT does not increase the risk for ASCVD.

## Discussion

To the best of our knowledge, this is the first clinical study to investigate the effect of WMT on dyslipidemia in Chinese patients, especially hyperlipidemia, indicating that the manipulation of intestinal microbiota may be a new method for the treatment of dyslipidemia. These data indicate that WMT has a lipid balance effect on patients with hyperlipidemia or hypolipidemia in the short and medium terms but has no improvement effect on the main outcomes of HOMA-RI, BMI, liver fat attenuation, and liver stiffness from short to long-term, which are the primary outcomes of this study. There was no significant difference between the low-risk and non-dyslipidemia groups, indicating that WMT would not increase the long-term risk for ASCVD and dyslipidemia, which are the secondary outcomes.

Many scientific evidences indicated the potential role of microbiota in influencing human health (20). FMT was not only limited in digestive system diseases, such as the *C. difficile* infection (CDI) (8, 21), irritable bowel syndrome (IBS) (22), and IBD (22) but also had improvement in CS (1, 23), neurologic, and psychiatric disorders (24). MS is often associated with an imbalance of the host microbiota and inflammation (25). Although increasing evidence from animal disease models has described the potential causal relationship between changes in gut microbiota and metabolic syndrome (26), there were limited data that supported the role of FMT in humans suffering from features of CS (27, 28).

Randomized controlled trials involving lipid status reported mixed results of metabolic parameters. For example, Kootte et al. (29) and Vrieze et al. (11) showed that FMT from lean donors to patients with obesity with metabolic syndrome could improve insulin sensitivity in the short to medium term (6 weeks), but no significant difference in the long-term (after 18 weeks). A total of three randomized controlled trials (3RCTs) with 76 patients were met the definition of hyperlipidemia (see *Data Collection* section) (11, 29–31). In the patients with hyperlipidemia in our study, the mean baseline of TC and TG were 11.3 and 90.0% higher than those of three RCTs, respectively, while the baseline of BMI and LDL-C were 28.1 and 4% lower, respectively. In conclusion, the study showed that the Chinese population have a lower BMI but higher TC and TG results than Westerners.

Fu et al. studied 893 patients from the LifeLines-DEEP population cohort (LL-D) to study the relationship between the microbiota and metabolic risk factors for cardiovascular disease. Studies have found that the intestinal microbiota, independent of age, gender, and host genetics, might support the potential of therapies altering the gut microbiome to control BMI and TG and HDL-C levels (32). In the three RCTs, FMT treatment was no benefit to reducing BMI, TG, HDL-C, and LDL-C levels of patients with obesity and MS (31). Our study showed that TC, TG, and LDL-C responded well to WMT treatment in the medium term. It is worth considering the similarities and differences of lipid profiles between the three RCTs, LL-D, and our study. First, our data provided real clinical evidence that supported the LL-D study, which showed that FMT has a better effect on reducing TC and TG in individuals with lower BMI. Second, pool-multiple feces and multiple treatments in a short time can improve the effect of FMT in our study and the study in recurrent CDI (33, 34). The diversity and richness of the gut microbiota supplied to the patients in the short-term may be an important factor in reducing blood lipids. Third, WMT is more effective in improving lipid metabolism disorder than manual FMT and oral capsule administration (35, 36). In addition, WMT was used to treat patients with dyslipidemia for three courses (each course was treated for three consecutive times) in our study. Different biological factors include a low BMI of 25 (similar to the Craven standard), as compared to other studies, and most of our patients had functional intestinal diseases and were Chinese.

Our results showed that WMT treatment could not only significantly reduce the blood lipid level of patients with hyperlipidemia but also increase the blood lipid levels of hypolipidemia to normal, significantly in the TC levels in the short and medium terms. The efficacy of WMT remained to be explored for long-term because of the small samples size. As to our data, there was no increased risk of blood lipid and AEs in people with normal blood lipid after WMT (37). We also found the same conclusion as Xi et al. that people with functional gastrointestinal diseases and dyslipidemia had no significant changes in BMI after WMT treatment (38).

The in-depth studies of intestinal function show that intestinal microbiota plays a key role in affecting systemic metabolism and other cardiac metabolic diseases (1, 39, 40). In Type 2 Diabetes Mellitus (T2DM) animal models, FMT can improve HOMA-IR and insulin sensitivity and repair damaged islets, providing a potential strategy for the treatment of T2DM (41–43). FMT has short-term or no benefit in improving insulin sensitivity and HOMA-IR in patients with obesity and MS (12, 29, 31, 36). In our hyperlipidemia data, HOMA-IR increased after WMT treatment from short- to long-term, but there was no significant change in HOMA-IR in the normal and hypolipidemic groups. We found no significant difference in liver fat attenuation and measurement of liver stiffness. The improvement of liver function cannot be concluded because the technical factors include the accuracy of the instrument and the limited number of patients for liver fat attenuation and measurement of liver stiffness. The relationship between FMT and hyperlipidemia on HOMA-IR needs to be further explored.

The increased risk for cardiovascular events is not only associated with dyslipidemia but also with abnormal glucose metabolism and liver function (44). Patients with T2DM have an increased risk for cardiovascular disease, and combination therapy consisting of metformin and statin are the commonly used treatments. However, both drugs act on glucose and lipid metabolism, which could lead to AEs when used in combination as compared to monotherapy. The percentage changes of lipid variables of patients during the combination therapy of metformin and atorvastatin/simvastatin were 10% to −31% in TC, 12% to −33% in TG, 1% to −36% in LDL-C, and 9% to −65% in HDL-C (45). Our data showed the percentage changes of lipid variables of patients after WMT treatment were TC (−5.2% to −9.5%), TG (−8.7% to −35.1%), LDL-C (−3.2% to −17%), and HDL-C (5.1% to −7.3%), respectively. The comparison of drug treatment and WMT treatment shows that WMT has a reasonable therapeutic effect on hyperlipidemia, especially in reducing TC.

Many factors affect the outcome of FMT, namely, donor selection and preparation, sample handling, mode of administration, and colonization resistance (1). FMT-related AEs are challenges in the application of FMT. In most cases, FMT was well tolerated with mild gastrointestinal AEs (46). Zhang et al. showed washed microbiota preparation by repeated centrifugation plus suspension for three times based on an automatic purification system that significantly reduced AEs (9). Our WMT program is based on Zhang’s standard. No serious AEs were found during or after WMT. Craven indicated that the improvement in small intestinal permeability was associated with allogenic WMT in patients with NAFLD and MS after WMT (12). In our study, patients who mainly received WMT had the characteristics of functional gastrointestinal disease. The results showed that the improvement of dyslipidemia was due to the improvement of intestinal permeability and gut microbiota after WMT. The results of clinical studies suggest that functional gastrointestinal diseases, WMT frequency, and BMI range are keys to the efficacy of WMT in the treatment of dyslipidemia. However, understanding the influence of the intestinal microbiota on metabolic diseases is in the initial stage, and data regarding the function of WMT on dyslipidemia are lacking. This is the first large-scale retrospective trial of dyslipidemia in China, which included hyperlipidemia, hypolipidemia, and normal blood lipid groups. We have established clinical evidence on the effect of WMT on lipid metabolism, which lays a foundation for the follow-up study of the effect of intestinal flora, metabolism, and human genome regulation on dyslipidemia. Furthermore, WMT may have potential bidirectional adjustment with lipid metabolism and may be a potential treatment for malnutrition. Taken together, these results suggest the beneficial effect of the microbiota in lipid metabolism; however, its mechanistic explanations need additional investigation.

This study has several limitations. Firstly, this study mainly focused on the analysis of clinical lipid and carbohydrate metabolism. Intestinal microbiota and metabolomics before and after WMT have not been evaluated. Therefore, the lowering effect of WMT on lipids and related microbiota remains unclear. Secondly, this is a single center study. A small number of patients returned to the hospital for long-term benefit evaluation of WMT treatment one month after the third treatment. Therefore, more data are needed to confirm the long-term efficacy of WMT in the treatment of dyslipidemia. Third, the risk factors of WMT on dyslipidemia, especially hyperlipidemia-related risk factors, may be insufficient. Fourth, we did not consider the potential confounding factors between the main symptoms for WMT treatment and blood lipids. Although the data show that WMT can improve dyslipidemia in the short and medium term, we should carefully interpret the research results of WMT and need large-scale prospective studies to further verify our conclusions. In the future, we plan to conduct a large sample prospective study to verify the effect of WMT on blood lipids.

## Conclusion

The retrospective analysis showed that WMT treatment changes blood lipids in patients with hyperlipidemia and hypolipidemia without serious AEs, whereas no risk was found in increasing blood lipids and ASCVD in the long-term. WMT treatment significantly decreased TC, TG, and LDL-C levels in the medium term for hyperlipidemia, but no significant difference in the long-term treatment. Therefore, the regulation of gut microbiota by WMT may indicate a new clinical method for the treatment of dyslipidemia.

## Data Availability Statement

The original contributions presented in the study are included in the article/supplementary materials. Further inquiries can be directed to the corresponding author.

## Ethics Statement

This study was conducted and approved by the Ethics Committee (No. 2017-98) in accordance with the Declaration of Helsinki at the First Affiliated Hospital of Guangdong Pharmaceutical University, Guangzhou, China. The patients/participants provided their written informed consent to participate in this study.

## Author Contributions

X-XH and FL designed the concept of the study. XL, ZD, and H-JZ collected and analyzed the data. H-HZ, WZ, and QL were the consultant for cardiology and pharmacology. FL and Y-LL wrote the draft manuscript. All authors listed have made a substantial, direct, and intellectual contribution to the work and approved it for publication.

## Funding

This study was supported by the Natural Science Foundation of Guangdong Province (2019A1515010125), the Department of Education of Guangdong Province (2019-GDXK-0013 and 2020KZDZX1132), and the Traditional Chinese Medicine Bureau of Guangdong Province (20201193).

## Conflict of Interest

The authors declare that the research was conducted in the absence of any commercial or financial relationships that could be construed as a potential conflict of interest.

## Publisher’s Note

All claims expressed in this article are solely those of the authors and do not necessarily represent those of their affiliated organizations, or those of the publisher, the editors and the reviewers. Any product that may be evaluated in this article, or claim that may be made by its manufacturer, is not guaranteed or endorsed by the publisher.
